# Medical Education in France

**Published:** 1878-09

**Authors:** T. K. Cruse

**Affiliations:** Paris, France


### Selection.

### MEDICAL EDUCATION IN FRANCE.

THE WAY MEDICAL APPOINTMENTS ARE SECURED — THE OVERCROWDED PROFESSION IN THE UNITED STATES, AND OUR MEDICAL COLLEGES. VAGINAL EXAMINATIONS IN PARIS. THE PAQUELIN CUATERY.

*To the Editor of the Medical Record :*

SIR:—If I had the wealth of a Vanderbilt or an Astor, I would advertise a free excursion ticket and a week in Paris to ten thousand of our medical men, on the sole condition that during that time they made themselves familiar with the system of medical education and appointments in vogue here, and witnessed a public examination. This examination, or *concours*, is the admirable machinery by which the results of prolonged application are determined and the rewards adjudged to the meritorious. A man needs no friends, no influence, provided he has brain-power. Let him work. Step by step he rises, until he reaches the goal —eligibility for appointment to one of the chairs of the *Ecole de Médecine.* This is the sole position to which candidates are directly appointed without previous immediate competition by *concours*, and possibly some wire-pulling may ultimately determine a choice. But here is an embarrassment of riches. The government is jealous of a school that has had for teachers such men as Orfila, Trousseau, Cruveilhier, Malgaigne, Berard, Nélaton, and Velpeau—jealous, I mean, of the repute and lustre of the past lights, and there is no chance, no possibility of an unworthy appointment. It is a precedent, so far as I know never violated, to take the new incumbent from the list of *professeurs agrégés*, and when one knows the innumerable tests to which it is necessary to submit before one is permitted to write *agrégé* after his name, it is certain that if wire-pulling is employed, it is because of the equally extensive attainments of those who are eligible. When men know everything about medicine, it is impossible to say that one candidate is more worthy than another.

My reason for desiring, as a possibility, a great number of American physicians to come and see the methods here is, that I doubt not some improvement will result therefrom to our own wretched system. Patriotism is all very well in its place, but I am not one of those representatives of the great republic who go about the Old World resolved to find nothing there in which America cannot lick all creation. Indeed, if I had not my hospital certificate to fall back upon, I doubt whether I would announce myself as the bearer of an American diploma. The youngster with his new parchment, certifying to his being a *virum probum*, and all the rest of it, proud of honors won by a diligent grubbing upon Neil and Smith's Compendium, will receive a great shock when he learns that he is suspected of having obtained his document by an equivalent of money and nothing else, for these Frenchmen know well that to obtain a *bona fide* diploma from the best New York schools, the money is the main thing, and consequently they hold his cherished possession in the most particular contempt. Are they not fully justified ? To graduate here, a native must first have his baccalaureate diploma. The School of Medicine does not even accord a matriculating examination to French students. The latter must first be prepared by general for professional study. There are five separate examinations to pass, and at the beginning of every third month the student must take an "inscription," that is, register his name at the bureau of the Faculty. After four inscriptions, he is allowed to come up for examination in chemistry, natural history and botany. After the twelfth inscription, the second examination takes place. This is in anatomy and physiology. At the end of the fourth year, the sixteen inscriptions having been made, pathology, hygiene, legal medicine, pharmacy, materia medica and therapeutics are the subjects for examination. The last examination—the fifth—is instituted at the Hôtel Dieu or La Charité. This is a practical test. The thesis amounts to something else than a rehashed chapter of a text-book. It is printed, and if exceptionally good, lays the foundation of a reputation for the author. Lastly, students serve in the hospitals, and go through the wards with the professors.

There is real clinical instruction—not merely talks to several hundred persons assembled in an amphitheatre. Let the newly-made M. D. of one of our New York schools ponder on these things, especially if he has taken two full courses; the first, at a winter college, the second, the ensuing spring, at one of those accommodating institutions specially organized so that a man can assume all the honors of graduation after paying his money—this always first—and attending, or strictly speaking, waiting during a course of lecturing of eight months. Medical men here know these things, and very properly decline to consider the product of our own ridicuously superficial and inadequate course worthy of any comparison with their thorough training of aspirants for a degree. Their own professors are men of eminence who have won their positions and are paid by the government; they know that in our numerous schools the professors appoint themselves, and by public advertisement and underbidding—such as is found in the circulars of some of our colleges, addressed to physicians and offering to discount the cost of a course to their students—compete for the prize of a large class. Here the only interest of the examiners is to determine the fitness of the candidate to assume the honors of his profession, and they rather enjoy plucking the unlucky dullard! With us self-interest, the money paid, and numerous considerations equally pertinent, combine to insure tender treatment at examinations, and the individual whose gross ignorance and stupidity compel his rejection at the hands of the more important school, can search further with the certainty of finding some impecunious "university" glad to render him full equivalent for his fees.

Here, when a hospital position is vacant, they do not load some well-known name with the additional title, or push a nonentity into the place, to the amazement of those who wonder by what elaborate machinery of wheels within wheels this kind of business is done while the seat is yet warm from the previous occupant. On the contrary, a general public announcement invites those who are entitled, by reason of many previous competitions, to hand in their names, accompanied by their contributions to medical literature, and a record of honors, past and present. This is but preliminary to a new and formidable test, and the decision is made by strict, impartial, skilled judges of the quality of brains—judges who themselves have proved, likewise by public *concours*, their fitness to sit in such a tribunal. These men have heard of our beautiful and simple process, known to the vulgar as *boosting*. They know how a man who walks on the stage at Steinway's, or the Academy of Music, to the music of the band, and after having been prayed over, to receive a dummy diploma with blue ribbons, may have invisible letters marking him as the special *protégé* of the faculty and the certain future incumbent of a vacant or specially-created chair. It is understood here that the favorite will readily obtain a dispensary position and a "professorship;" that next he will be found airing his anatomy as a demonstrator, and as the proprietor of a favored "quiz;" that, *presto!* the spring is touched, and the humble set of poor practitioners who knew him as a classmate—then not distinguished for anything in particular—look up to him standing on the dizzy heights of a full professorship. Here there are complaints of the overcrowded state of the profession in France, yet the bulletin board of the medical school contains at the present moment no less than ten different appeals from this or that mayor begging a doctor to come and settle in his locality, guaranteeing board, providing the indispensable *voiture*, and offering a stipend of from one to three thousand francs—this sum being over and above the receipts from practice. If a man chooses to work he is absolutely sure of success. There are numerous positions, salaried and desirable, open to any member of the French faculty; but for these there is first the inevitable *concours*.

Is it possible for us in America ever to have a better system? It is only by personal appreciation of the way medical matters are managed here and in Germany, even in England, that the shame and scandal of the way of doing similar things at home force themselves with effect upon the observer. This subject can never be old or trite until the disinterested part of the profession take matters in their own hands, and not merely demand but work reform. Here the profession is under governmental supervision, in governmental pay; a multitude of venerable forms appertain to it, and the machinery works with the ease and noiselessness that results when time wears away all points of friction. It is as idle as undesirable to wish for legislation on the subject in the United States. Congress has no proper concern with us, but reform is the proper function of the body of intelligent young medical men who must know and feel the disgrace that attaches to us from a system of education that permits a ploughboy or a day laborer, with the one single and only indispensable requisite of a little money, to take his seat on a bench to hear lectures on subjects the very terms of which fall on his ear as the words of a foreign language, and, after eight months—possibly of continuous real or nominal attendance—to be turned loose on society to practice on its infirmities, and to talk in bad English virtuously and indignantly against the quacks. Besides, it is against all reason that the annual spring tides of M. D.'s, fledged by the patent incubating process, are thrown, year after year, on the community, to diminish still further the absurd figure, 600, expressive of the number of people to which there is one physician. Who is benefited by this host of annual accessions—the hosts themselves? No. The body of the profession? How many times has it happened that a new-comer in a locality settled and was doing fairly, with a constituency just sufficient to keep him so, when the next year one, two or three fresh aspirants, direct from college, came to the neighborhood, spoiled his own business, and went on starvation rations themselves? Is the community at large benefited by new accessions to our ranks? In one respect only: the cheat, whose credit is exhausted everywhere else, can always find a physician to serve him without fee. Who then remains to be benefited by the spring hordes? The thousand professors who compose the faculties of the private speculations known as colleges. It seems that for these favored individuals everybody else must suffer. Many of these institutions are rich. They control all the patronage, so that necessarily eminent men belong to them as teachers. To speak the truth about them is not the way to preferment, but the majority of us had better make up our minds that we can't all be professors, and try for something that united action will make attainable.

I started to write about Paris medical matters, and have gone off riding a hobby instead. The fact that hospitals have their clinics and shows at eight or nine o'clock in the morning makes the effort to attend bad for lazy people. Fortunately I am located near that model of hospital structure, the new Hôtel Dieu, and most of my sight-seeing has been there. Richet, the crack surgeon, is getting old, and shows much shining pate. I never saw a man examine a case more thoroughly. Jolly little Kusco, or Cusco, him of speculum fame, I believe, handles women in a way that would excite execration at home, and which I can't believe is proper anywhere. To expose women from their feet to their breasts, without the comforting pretense of a sheet, and to punch their abdomens as if he was going to dig the uteri out through the walls, is not the way to make the examination chair a joy and delight. I saw one young woman of some personal attractions, and who seemed to have some modesty left, although she was a low-class French woman, tremble as if in ague during the ordeal, and shriek with pain when he forced his very short fore-finger up to the cervix as if he intended to carry all before him. The benzine cautery is much used here. The most painful operation I ever saw without an anæsthetic was a sarcoma removed from the root of the neck. An incision was made around the base of the tumor, save that two little isthmuses of skin were left above and below until the growth had been severed from its connections beneath. These bands of tissue were burned across, and the vessels cauterized at the same time. Lastly, the islands of skin were *burned* through. Save this last feature, the operation struck me as just the procedure for dangerous situations. But it had an unpleasant suggestion of the time they amputated with red-hot knives, and Ambrose Paré, could he have seen it, would doubtless have been scandalized at the abandonment of the ligature.

The most striking thing to a New Yorker about this hospital is the building itself. Save that palace for paupers—the new St. Thomas' hospital in London—the Hôtel Dieu is unquestionably the finest structure of the kind in Paris or London. It contains at present only six hundred beds. A number are yet to be added, but it covers a much larger space than Charity or Bellevue, and in appointments and service is only rivaled by the New York Hospital. I suppose our commissioners would give up the ghost if it were proposed to put up a building for a public hospital with the corridors and two immensely long covered galleries of fine dressed stone, without a suspicion of plaster; where each one of a long line of stone columns must have cost a large sum; where a chapel of really tasteful architecture looks as solid as if it had been taken bodily from a part of *Notre Dame;* where a steam engine runs day and night to fan fresh air into the wards; where every appointment is of the most costly description; and where, last but not least, the patients are addressed as *messieures* and *mesdames les malades.*

PARIS, France. T. K. CRUSE.

---

A FABLE.—A black Crow, with a white neck-tie and gold Eyeglasses, was once perched on a Limb of the Law of Ethics, having in his beak a Big piece of Cheese, wrapped in a Newspaper containing his Advertisement. At the foot of the tree were more than a dozen Foxes who Professed Medicine.

"Pray let us hear your superb voice," chorused the latter. "Most willingly," responded the Crow; and he unwrapped the Cheese, which he carefully deposited upon another Limb of the Law, and threw down the Newspaper, which the Foxes at once devoured for the sake of the scent of the Cheese which still hung round It. This done, the Crow favored his auditors with the popular ballad: "It's naughty but it's nice!" and, after having flung some Brazilian pebbles into the crowd, he quietly enjoyed his Cheese.

*Moral.*—For a fracture of the Code, continued "extension" is bad, and the Newspaper splint worthless. The best dressing is *Cheese à la Brazilienne.*

ÆS OP(TIC).

